# Comparison of the Vitreous Fluid Bacterial Microbiomes between Individuals with Post Fever Retinitis and Healthy Controls

**DOI:** 10.3390/microorganisms8050751

**Published:** 2020-05-17

**Authors:** Kotakonda Arunasri, Malleswarapu Mahesh, Gumpili Sai Prashanthi, Rajagopalaboopathi Jayasudha, Sama Kalyana Chakravarthy, Mudit Tyagi, Rajeev R. Pappuru, Sisinthy Shivaji

**Affiliations:** 1 Jhaveri Microbiology Centre, Prof Brien Holden Eye Research Centre, L V Prasad Eye Institute, L V Prasad Marg, Banjara Hills, Hyderabad 500034, India; drarunasri@lvpei.org (K.A.); maheshmalleswarapu9@yahoo.com (M.M.); prashanthi9.nitw@gmail.com (G.S.P.); jayasudha001@gmail.com (R.J.); samakalyana.lvpei@gmail.com (S.K.C.); 2 Smt. Kanuri Santhamma Centre for Vitreo Retinal Diseases, L V Prasad Eye Institute, L V Prasad Marg, Banjara Hills, Hyderabad 500034, India; drmudit@lvpei.org (M.T.); rajeev@lvpei.org (R.R.P.)

**Keywords:** metagenomic sequencing, vitreous of eye, post fever retinitis, bacterial microbiome

## Abstract

Ocular microbiome research has gained momentum in the recent past and has provided new insights into health and disease conditions. However, studies on sight threatening intraocular inflammatory diseases have remained untouched. In the present study, we attempted to identify the bacterial microbiome associated with post fever retinitis using a metagenomic sequencing approach. For this purpose, bacterial ocular microbiomes were generated from vitreous samples collected from control individuals (VC, *n* = 19) and individuals with post fever retinitis (PFR, *n* = 9), and analysed. The results revealed 18 discriminative genera in the microbiomes of the two cohorts out of which 16 genera were enriched in VC and the remaining two in PFR group. These discriminative genera were inferred to have antimicrobial, anti-inflammatory, and probiotic function. Only two pathogenic bacteria were differentially abundant in 20% of the PFR samples. PCoA and heatmap analysis showed that the vitreous microbiomes of VC and PFR formed two distinct clusters indicating dysbiosis in the vitreous bacterial microbiomes. Functional assignments and network analysis also revealed that the vitreous bacterial microbiomes in the control group exhibited more evenness in the bacterial diversity and several bacteria had antimicrobial function compared to the PFR group.

## 1. Introduction

Several ocular manifestations like conjunctival congestion, uveitis, episcleritis, neuroretinitis, dacryoadenitis, and retinitis [1] have been reported to manifest following acute systemic febrile illness. Theses manifestations are not dependent on the etiology of the systemic fever and are not related to whether the fever was due to either bacteria, viruses, or protozoa. Postfever retinitis (PFR) is also one such retinal inflammatory disorder that usually manifests between two to four weeks post systemic fever irrespective of the etiology [1]. In PFR, posterior regions of the eye are affected and clinically patients present with focal and multifocal patches of retinitis which could be unilateral or bilateral, possible optic nerve involvement, serous detachment at the macula, macular edema and localized involvement of the retinal vessel in the form of beading of the vessel wall, tortuosity, and perivascular sheathing [2]. A small proportion of PFR cases are also infectious in etiology and are caused by bacteria ((*Mycobacterium tuberculosis* (Tuberculosis), *Salmonella typhi* (Typhoid), *Leptospira* spp. (Leptospirosis), *Rickettsia* spp. (Rickettsial retinitis)), protozoa (*Toxoplasma gondii* (Toxoplasmosis)), and viruses [3,4,5,6,7,8]. However, a significant number of PFR cases are due to an unknown etiology and have been attributed to the compromised immune status in the affected individuals. A characteristic feature of PFR is that it usually manifests between two and four weeks after the fever in immunocompetent patients and patients present with sudden and painless onset of diminution of vision [1,2]. Despite all these clinical manifestations, it has always been a challenge to identify causative agents if there are any associated with PFR.

Routine culture and PCR based methods have failed to identify microorganisms associated with the vitreous of PFR individuals [9]. This failure to detect microbes in the vitreous of postfever retinitis does not necessarily prove the absence of microorganisms. It could also imply that the microorganisms are present but are not amenable to the routine PCR or culture methods. Thus one may have to employ a more refined and sensitive method for detection of the microorganisms like using the advanced technique of Next Generation Sequencing (NGS). In this culture independent approach, the metagenome from the sample comprising the genomes of bacteria, fungi, and viruses would be sequenced and subjected to phylogenetic analysis to identify the diversity of microbes [10]. NGS has been used earlier to successfully identify microbes associated with diseases like endocarditis in which conventional methods failed to identify the causative agent (*Streptococcus gordonii*, *S. oralis*, *S. sanguinis*, *S. anginosus*, *Coxiella burnetii*, and *Bartonella quintana*) [11].

In the present study NGS was employed to understand the microbial composition in the vitreous of healthy controls and individuals with postfever retinitis. This approach would be helpful in identifying specific microorganisms present, if any, in the vitreous of patients and would also be useful for understanding the changes in the microbiome due to the prevailing inflammatory conditions. The present study would mainly focus on the alterations in bacterial microbiomes in the vitreous of individuals with postfever retinitis (PFR) compared to the vitreous of the control group (VC).

## 2. Materials and Methods

### 2.1. Sample Collection

Vitreous fluid was collected by vitreous biopsy/pars plana vitrectomy from PFR individuals (*n* = 9) (Table S1) following standard indications for the planned management of the patients by the ophthalmologist. Vitreous samples collected from individuals undergoing macular hole surgery or rhegmatogenous detachment (*n* = 19) served as controls (VC) since these individuals had no PFR and no other ocular or systemic indication (Table S1). Sample size was derived by the population proportion method. The parameters set for deriving the size included 90% confidence interval and a 5% margin of error. Participants who had inflammatory disorders of the eye, uncontrolled glaucoma, hypertension, and diabetes were excluded from the study. Approximately 300 µl of vitreous fluid was aspirated from each eye and stored at −80 °C until use. Institutional review board (LV Prasad Eye Institute, Hyderabad) approved the study (LEC 09-17-079). The study design adheres to the tenets of the Declaration of Helsinki.

### 2.2. DNA Extraction and Sequencing of the Samples

Vitreous fluid (200 µL) was used to extract DNA using PureLink DNA extraction kit (ThermoFisher Scientific, Mumbai, India). Quality of the DNA was judged by gel electrophoresis on a 1.0% agarose gel and the concentration was quantified using a Qubit 3.0 fluorometer (Thermo Fisher Scientific, Carlsbad, CA, USA). The extracted DNA was used for subsequent metagenome analysis. For this purpose, the total nucleic acids were amplified with random hexamers using amplification kit (SeqPlex, Sigma Aldrich Chemicals Private Limited, Bengaluru, India). The DNA was then processed for library preparation and sequenced according to the NEBNext Ultra DNA Library Prep Kit for Illumina Nextseq 500 PE sequencing protocol. Sequencing was performed on the Illumina Nextseq 500 platform using paired-end sequencing with 2 × 150 bp chemistry. In addition, at every stage of sample preparation, during DNA extraction, PCR reactions and whole genome amplification, sterile water was used as negative control to ascertain the possibility of bacterial contamination from the environment. However, PCR was consistently negative for amplification of DNA from water and the amplification mix without DNA which served as the negative control compared to samples. No sequences could be generated from the negative controls.

### 2.3. Whole Metagenome Analysis

The raw sequence reads from all the samples were analysed for identification of microorganisms. FASTQ files of the raw reads were analysed for quality parameters like read length, Phred score, GC content, and ambiguous bases. Subsequently, adapters were trimmed using trim-galore program (version 0.4.0; Felix Krueger and Cutadapt version 1.2). Post-trimming, reads were subjected to QC using FastQC (version 0.11.3). More than 90% of the data passed Q25 quality score, which was further used for downstream analysis. Sequences were then aligned with human reference genome (GRCh38) to remove human genome sequences and then the unaligned reads were recovered. Background sequences due to run processing were also filtered from the recovered unaligned reads. The unaligned reads were then assembled (denovo) using metaSPAdes genome assembler (Version 3.12.0) into contigs which were subsequently analysed using RAPSearch. RAPSearch is a tool for protein similarity search for short reads against nonredundant protein database from National Center for Biotechnology Information (NCBI). Meta Genome Analyser (MEGAN v 5.11.3) was used to identify the diversity for taxonomy assignment and KEGG pathway analysis.

### 2.4. Statistical Analysis

The vegan package in R (http://vegan.r-forge.r-project.org/) was used to rarefy the 28 bacterial microbiomes and to quantify alpha (Shannon diversity, Simpson index, and Observed number of genera) and beta diversity indices. Genera showing a mean abundance >0.002% were used for the analysis. Wilcoxon signed rank test (with *p* < 0.05 as significant) was carried out to identify significantly different taxa in both VC and PFR group bacterial microbiomes at phylum and genus (discriminative genera) level. In addition, linear discriminant analysis effect size (LEfSe) was applied to identify discriminant bacterial genera. For this, the genera of VC and PFR groups were subjected to a nonparametric factorial Kruskal–Wallis (KW) sum-rank test *p* <0.05, LDA > 2.0 (http://huttenhower.sph.harvard.edu/galaxy/). Further, the statistical significance of the differences of the discriminative genera for VC and PFR group was tested by one-way ANOVA followed by t- test (*p* < 0.05). In the vitreous differentially abundant KEGG, functional pathways between VC and PFR were also ascertained using Wilcoxon signed rank test (with *p* < 0.05 as significant). Principal Coordinate analysis (PCoA) plot was generated for the 28 bacterial microbiomes using ade4 package in R (v 3.2.5). Jensen–Shannon divergence was used as a distance metric (http://enterotyping.embl.de/enterotypes.html). VC and PFR clusters on the PCoA plot were identified by subjecting the data to K-means clustering (k = 2) [12].

### 2.5. Correlation Network Analysis of Bacterial Genera

CoNet [13], a Cytoscape [14] plugin, was used to detect interactive networks of the bacterial microbiome of VC and PFR groups independently. The differentially abundant genera of VC and PFR groups were used to analyse the bacteria–bacteria interactions at the genus level based on pair-wise correlations between abundances using Spearman correlation coefficient (r). To build the network, the genera were filtered with frequencies less than 0.05.

## 3. Results

### 3.1. Bacterial Microbiomes in the Vitreous of Control and Postfever Retinitis Groups

**Figure 1:**
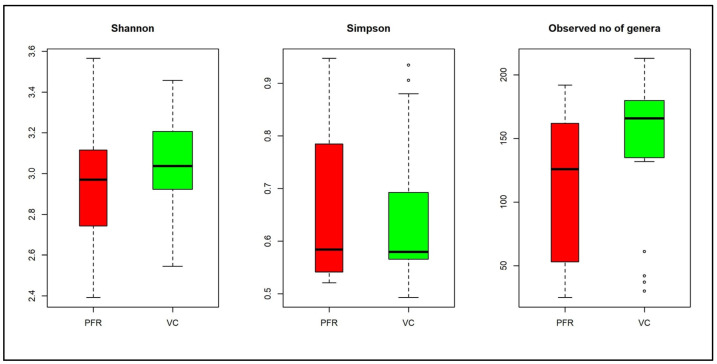
Box-plots depicting the Alpha diversity indices in the vitreous fluid of control (VC) and postfever retinitis (PFR) groups. Median values (horizontal line) and interquartile ranges have been depicted in the plots.

**Figure 2:**
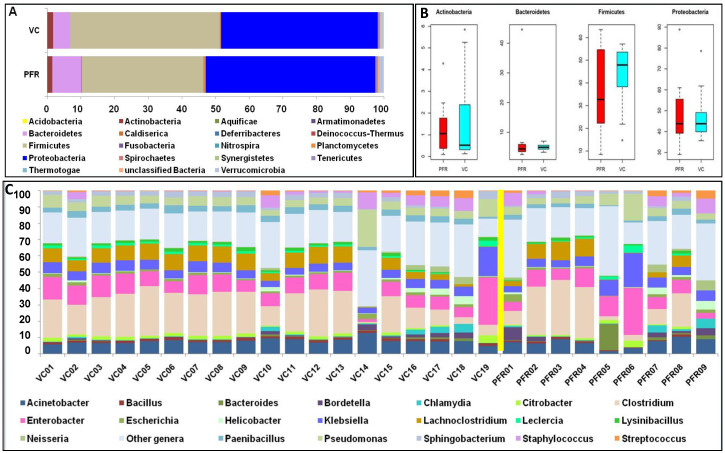
**Relative abundance of bacterial phyla and genera in the vitreous fluid of VC and PFR individuals.** Abundance of different bacterial phyla (**A**,**B**) and bacterial genera (**C**) in the vitreous fluid of control (VC) and postfever retinitis (PFR) individuals (The yellow line in “C” separates VC and PFR groups. Median values (horizontal line) and interquartile ranges have been depicted in the plots.

Whole metagenomes were generated from the vitreous fluid of controls (VC, *n* = 19) and Postfever retinitis (PFR, *n* = 9) individuals (Table S1). A total of 126.64 million reads (Phred score > 25) were generated for all the 28 vitreous samples with an average of 4.52 million reads per sample (Table 1). Out of these reads, a total of 1.14 million reads were assigned to bacteria and the average number of reads assigned to a bacterial microbiome was 40,753 (Table 1). Rarefaction curves were plotted for all the bacterial microbiomes and most of the samples showed tendency towards saturation, indicating that sufficient depth and coverage was achieved (Figure S1). Alpha diversity indices (Simpson, Shannon, and Observed number of genera) showed differences between the two groups (Figure 1). Taxonomic assignment and hierarchical classification of the reads revealed that a total of 18 bacterial phyla were detected in both VC and PFR groups (Figure 2A). The number of phyla common to both VC and PFR groups were 12 and the number of phyla specifically detected in VC and PFR groups was 14 and 16 respectively. Unclassified reads accounted for a mean abundance of 0.29% and 0.38% in VC and PFR groups respectively (Table S2). Phyla *Firmicutes* and *Proteobacteria* were predominantly present in all the 28 samples (Table S2). The percentage mean abundance of phylum *Firmicutes* was 44.52 in VC and 36.3 in PFR samples. The percentage mean abundance of phylum *Proteobacteria* was 46.57 in VC and 50.33 in PFR samples (Table S2). The visible differences in the mean abundances of phyla *Firmicutes* and *Proteobacteria* were not significant in PFR compared to VC group (*p* > 0.05) (Figure 2A,B). Further, the number of reads of *Bacteroidetes* and *Proteobacteria* were slightly high in PFR samples compared to VC samples (Figure 2A,B).

### 3.2. Differentially Abundant Bacterial Genera in the Vitreous Fluid of Controls and Postfever Retinitis Individuals

**Figure 3:**
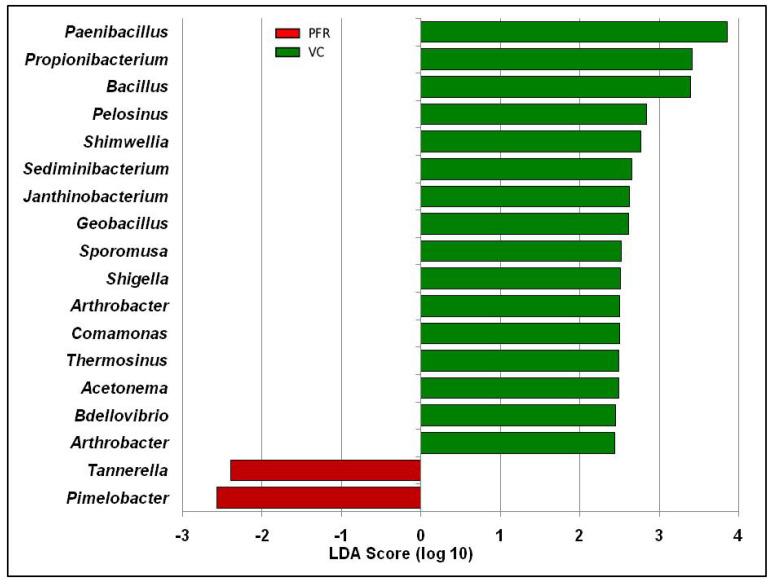
Linear discriminant analysis of the bacterial microbiome of VC and PFR groups. The bars in the figure represent the statistically significant genera as determined by the linear discriminant analysis (LDA) combined with effect size measurements (LEfSe). Green and red colour bars indicate increase in the relative abundance of the genera in VC group and PFR group respectively.

**Figure 4:**
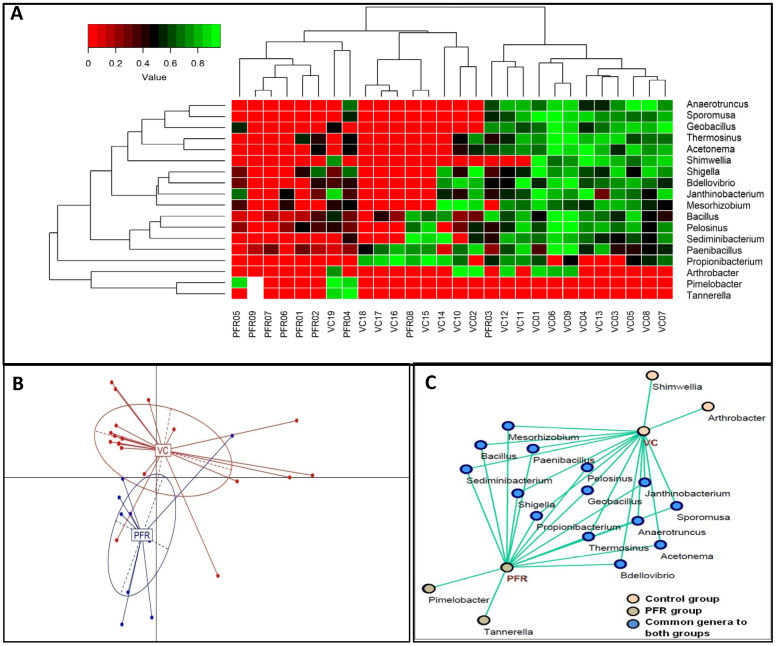
Discrimination of the vitreous fluid bacterial microbiomes of VC and PFR groups (**A**). Two dimensional heatmap showing rank normalized abundances (scaled between 0 and 1) of 18 discriminative bacterial genera in the control group (VC) and postfever retinitis (PFR) group (**B**). Principal coordinate analysis in controls (VC) and postfever retinitis (PFR) groups. (**C**). Correlation network of 18 discriminative genera in VC and PFR groups. Genera having a mean abundance > 0.002% were analysed.

The above reads could be assigned to 301 genera across all the microbiomes (Figure 2C and Table S3A,B) with 291 and 281 genera in the VC and PFR microbiomes respectively. Nearly 50% of the reads were assigned to five genera (*Clostridium*, *Enterobacter*, *Acinetobacter*, *Klebsiella*, and *Lachnoclostridium*). It was also observed that out of the 301 genera, 18 genera were differentially abundant in PFR individuals compared to VC (*p* < 0.05) (Table 2). In addition, linear discriminant analysis (LDA) combined with effect size measurements (LEfSe) analysis also showed 18 genera as significantly different between VC and PFR groups. Sixteen genera were relatively abundant in VC group and two genera were relatively more abundant in PFR group (Figure 3). A heatmap of the 18 discriminative bacterial genera separated VC and PFR vitreous fluids into two clusters. The majority of the VC (18 of 19) and PFR (7 of 9) microbiomes formed a separate cluster (Figure 4A). Principal co-ordinate analysis of the discriminative genera separated all the VC and PFR microbiomes into two distinct clusters (Figure 4B). It is worthwhile to mention that the PFR group also included three individuals viz., PFR07, PFR08, and PFR09 who were identified as having Retinitis and not PFR but nevertheless in the principal co-ordinate analysis they formed a single group, implying that the microbiomes of PFR and Retinitis are similar.

**Figure 5:**
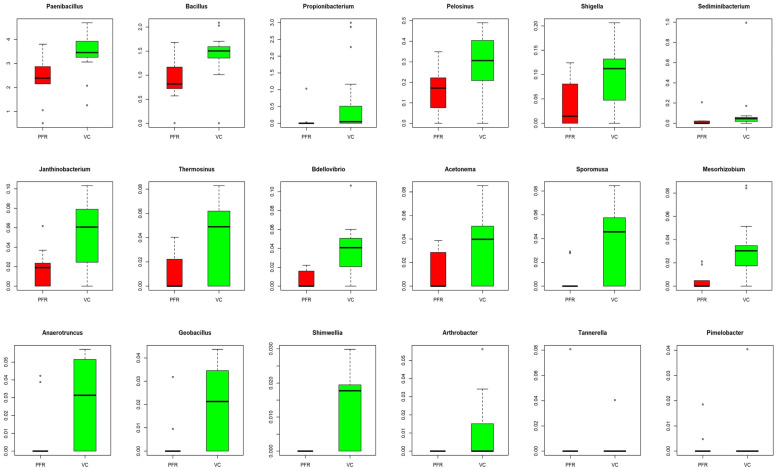
Box-Plots illustrating the differential abundance of genera in VC group compared to PFR group (*p* < 0.05). Median values (horizontal line) and interquartile ranges have been depicted in the plots.

A correlation network was constructed using Cytoscape for the discriminative genera of VC and PFR individuals which would reveal the interconnections among the genera of the two groups. The correlation network revealed that 14 genera were common to both VC and PFR groups and included *Anaerotruncus*, *Acetonema*, *Bacillus*, *Bdellovibrio*, *Geobacillus*, *Janthinobacterium*, *Mesorhizobium*, *Paenibacillus*, *Pelosinus*, *Sediminibacterium*, *Shigella*, *Sporomusa*, and *Thermosinus*. The network analysis also showed that two genera, namely *Arthrobacter* and *Shimwellia*, were present only in the VC group and absent in the PFR group. Similarly, two bacterial genera viz., *Pimelobacter* and *Tannerella* were exclusively present in PFR group but absent in VC group (Figure 4C). Box-plot analysis, a nonparametric test, was employed to interpret the differential abundance of the 18 discriminative genera in both VC and PFR groups (Figure 5). The analysis revealed that the mean abundance of 16 discriminative genera that includes 14 genera that were common to both the groups and two genera that were unique to VC were more abundant in control group compared to PFR group.

### 3.3. Interactive Network of Bacterial Genera in Control and PFR Group

**Figure 6:**
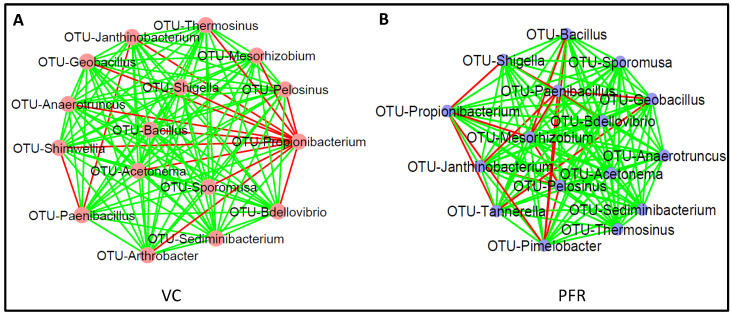
Bacterial microbiome interaction networks in VC (**A**) and PFR (**B**) constructed by CoNet using cytoscape. Nodes indicate the bacterial genera. Green edges indicate positive interaction and red edges indicate negative interaction.

Co-occurrence network analysis (CoNet) was done using the discriminative genera between VC and PFR groups separately (Figure 6A,B). In both VC and PFR networks, interactions were generated for 16 of the 18 discriminative genera. In VC group, Genus *Propionibacterium* which was present in 73.7% of the samples showed negative interactions with 13 other genera and positive interactions with only two genera. In contrast, the genus *Sediminibacterium* showed positive interactions with all the remaining 15 genera analysed (Table S4A). In contrast, in PFR group genus *Paenibacillus* showed maximum negative interactions (6 of the 18 genera). Five other genera viz., *Acetonema*, *Anaerotruncus*, *Sediminibacterium*, *Sporomusa*, and *Thermosinus* showed positive interaction with all the other genera (Table S4B). Remaining genera showed one or two negative interactions with other genera.

### 3.4. Kyoto Encyclopedia of Genes and Genomes (KEGG) Pathways Analysis

**Figure 7:**
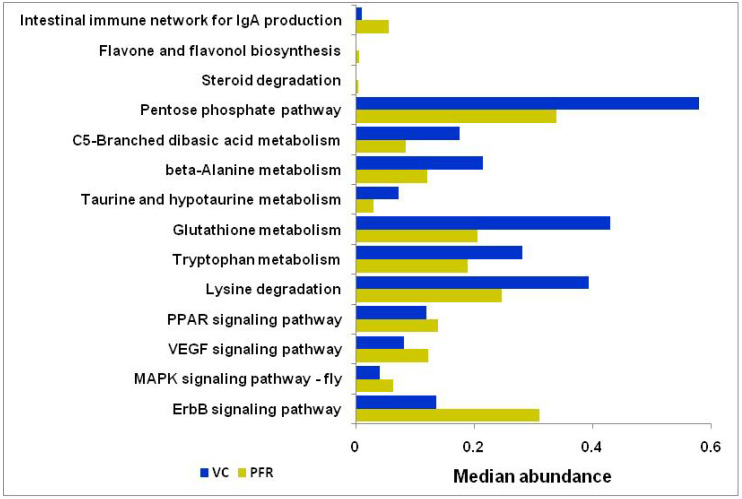
Differential abundance of KEGG pathways associated with PFR group compared to control group.

KEGG pathway analysis of VC and PFR groups determined enrichment of functions related to infectious diseases, immune system, and signal transduction. Willcoxon signed rank test of the enriched KEGG pathways identified statistically significant pathways in PFR compared to VC group which included viz., ErbB signaling pathway, MAPK signalling pathway-fly, VEGF signaling pathway, PPAR signaling pathway, flavone and flavonol biosynthesis pathways, steroid degradation pathway, and intestinal immune network for IgA production (Table S5 and Figure 7). It was also observed that the amino acid metabolic pathways such as Lysine degradation, Tryptophan metabolism, beta-Alanine metabolism, Glutathione metabolism, Taurine and hypotaurine metabolism, Pentose phosphate pathway, and C5-Branched dibasic acid metabolism were enriched in VC group compared to PFR group (Figure 7).

## 4. Discussion

Ocular microbiome studies that uncover microbial communities of the eye have revealed that the eye hosts a unique assemblage of microbes [15,16]. For instance, the ocular surface microbiome has a preponderance of commensal microbes which are different from the skin and oral microbiome [17,18]. Studies on intraocular fluids like the aqueous and vitreous are scarce and these fluids have been considered to be sterile but may host pathogen invaders during disease conditions [19]. Diagnosis of these intraocular pathogen invaders is a challenge and it is further complicated by the inability to culture the microbes using conventional methods and PCR based methods are often unsuccessful. However, a possible approach to overcome this challenge would be to resort to metagenome deep sequencing of the ocular fluids. This approach has indeed demonstrated that vitreous fluids from Endophthalmitis patients [19,20] hosts a variety of microbes which were otherwise undetectable using routine diagnostic approaches. However, Deshmukh et al., [20] using V3 and V4 targeted metagenome sequencing approach could not generate bacterial microbiome in control vitreous fluids, while Kirstahler et al. [17] using whole metagenomic sequencing approach could detect bacterial microbiomes in control vitreous fluids. Such studies on the identification of intraocular microbes could form the diagnostic basis for intraocular inflammatory diseases like uveitis and Age-related Macular Degeneration (AMD) [21]. In the present study, based on metagenomic sequencing of the vitreous fluid of VC and PFR individuals we demonstrate alterations in the bacterial microbiome in the PFR individuals. These findings may have implications in disease outcome (Figure 2A–C).

Studies on gut microbiome associated with systemic human Ulcerative Colitis, systemic sclerosis [22,23], and ocular diseases such as Keratitis [12,24], Uveitis [25,26,27,28], AMD [29,30], dry eye disease in SjÖgren syndrome patients [31] consistently indicated that the overall diversity and abundance of bacterial microbiomes was decreased in the disease condition compared to control group. In contrast, the ocular microbiomes showed an increase in the diversity in the diseased condition [32]. In agreement with this, the Alpha diversity indices (Simpson and Shannon) (Figure 1) did exhibit an increased trend in PFR group compared to VC group (Figure 1B). The Simpson index and observed number of genera indicated that the bacterial communities in VC compared to PFR group were distributed more evenly and thus could be more functionally stable compared to PFR group. This also means that the VC group shares more common taxa among the samples compared to samples within PFR group.

Gut microbiome studies in patients with ocular disease have shown that *Firmicutes* and *Bacteroidetes* were the predominant phyla irrespective of the disease condition [12,24,27,29]. In contrast, the ocular surface bacterial microbiomes showed that *Firmicutes* and *Actinobacteria* were present as the predominant phyla [33]. In comparison, the intraocular microbiomes in the present study showed phyla *Firmicutes* and *Proteobacteria* as the major phyla in both control and PFR groups (Figure 2A), thus confirming the recent findings of Deshmukh et al. [20]. Even at the genera level 10 genera viz., *Actinobacter*, *Bacillus*, *Enterobacter*, *Escherichia*, *Klebsiella*, *Nieisseria*, *Paenibacillus*, *Pseudomonas*, *Staphylococcus*, and *Streptococcus* that were shared between the control and PFR samples were also identified earlier in the endophthalmitis samples [20]. We also demonstrate that the VC and PFR groups share 272 genera (Figure S2) suggesting that 90% of the genera are shared by both the groups and only 10% of the genera are unique.

Previous cultivation-based assessments as well as metagenomic sequencing based analysis indicated that vitreous body of the eye is sterile and contains only a few microbial cells in individuals without eye infection [19]. Therefore, the mere identification of the bacterial genera in these samples does not imply association with ocular disease and it is important to perform appropriate statistical analysis to identify the specific organisms associated with PFR of the eye. Our results indicated that the bacterial microbiomes of the vitreous fluids of control and PFR groups are significantly different (*p* < 0.05) (Table 2). Further that the bacterial microbiomes of VC and PFR groups are distinct was also confirmed by correlation network and interaction network analysis of the 18 discriminative genera (Figure 4C). Functional analysis indicated that 11 of the 18 discriminative genera could be assigned to a physiological function (Table 3). When the abundance of these 11 genera was compared it was observed that in PFR, three probiotic genera (*Anaerotruncus*, *Arthrobacter* and *Shimwellia*), four antimicrobial genera (*Bacillus*, *Bdellovibrio*, *Janthinobacterium*, and *Paenibacillus*), two proinflammatory genera (*Propionibacterium and Shigella*) and one anti-inflammatory genus (*Sporomusa*) were decreased in PFR compared to VC. One proinflammatory genera (*Tannerella*) was also increased in PFR. Decrease in genera with antimicrobial and anti-inflammatory properties and increase in proinflammatory would support PFR. What could not be explained is the increase in anti-inflammatory genera. Earlier studies had indicated that the genus *Tannerella* is associated with pathogenicity [34,35]. The high abundance of antimicrobial and/or anti-inflammatory organisms in intraocular control samples compared to PFR group could imply that positive interactions of these genera would help in maintaining intraocular homeostasis in the vitreous fluid of controls. Co-occurrence network analysis depicts polymicrobial interactions (both positive and negative interactions) which could help in understanding the role of microbiomes in health and disease [36]. Microbes that exhibit positive interactions are likely to be more beneficial than those that exhibit negative interaction. In the VC group all the genera except *Propionibacterium*, an opportunistic pathogen with proinflammatory activity [37,38], exhibited more than 13 negative interactions out of 15 possible interactions with other genera. An earlier study had also indicated that *Propionibacterium* was associated with intraocular inflammatory diseases like Uveitis and Endophthalmitis [39]. Therefore, increase in the abundance of *Propionibacterium* in VC group was not anticipated. However, it is worthwhile considering the possibility that instead of *Propionibacterium* negatively influencing the 13 genera it is equally possible to consider that all these 13 genera in VC were together suppressing the negative effects of *Propionibacterium.* This indeed may be the case since these genera also possibly interacted with each other. Thus, based on the network analysis it appears that the bacterial microbiome in control group with multifaceted interactions could substantially minimise the activity of the proinflammatory bacteria and thus would maintain the bacterial microbiome homeostasis. While in the PFR group *Propionibacterium* had less negative interactions with other genera compared to control group. This may suggest that the bacterial diversity in PFR group has less protective function against proinflammatory bacteria compared to control group.

KEGG pathway analysis showed enrichment of 14 pathways with functions related to metabolic pathways and signal transduction in VC and PFR group. Amino acids serve as the metabolic mediators for the cross talk between host and the microbiome and thus the amino acid metabolic pathways would differ at the site of infection compared to the healthy state [48]. In PFR group the decrease in abundance of amino acid pathways such as those encoding for the metabolism of lysine involved in the survival of bacteria [49], Taurine, hypotaurine [50], glutathione [51], and tryptophan [52] involved during infection, showed low abundance in PFR group compared to control group implying the measure taken by the host cells to avoid infection. Four signalling pathways were also enriched in PFR group compared to control group (Figure 7). This enrichment was expected because these pathways are involved in infection like Vascular endothelial growth factor (VEGF) pathway which facilitates interaction between pathogens and host cells [53], the mitogen-activated protein kinase (MAPK) which is important in modulating host and pathogen interactions [54], epidermal growth factor receptor (EGFR) which modulates cell survival, proliferation, differentiation, and migration/invasion [55], and Peroxisome proliferator-activated receptor (PPAR) which gets activated during bacterial infection [53]. The enrichment of all these pathways in PFR may also be associated with the secretions of inflammatory cytokines and host cell apoptosis in response to retinitis in PFR group.

The major limitation of the study was recruiting healthy individuals as controls for the study. Therefore, vitreous fluid collected from individuals undergoing ophthalmic surgery for macular hole or rhegmatogenous detachment were used in the study. The other limiting factor is that PFR is a very rare ocular disease and therefore recruitment of patients is very time consuming and requires ethical compliance for vitreous biopsy.

## 5. Conclusions

The study confirms the presence of bacteria in the vitreous fluid.

(i) Demonstrates that the bacterial microbiomes of vitreous fluid from VC and PFR individuals could be discriminated at the genera level.
(ii) The vitreous fluid of VC individuals showed increase in abundance of anti-inflammatory and antimicrobial genera and decrease in proinflammatory genera.
(iii) The vitreous fluid of PFR individuals showed decrease in abundance of anti-inflammatory and increase in proinflammatory genera.
(iv) KEGG pathway analysis identified significant increase in signalling pathways in PFR group that are associated with inflammatory cytokines.

## Figures and Tables

**Table 1:** Number of reads assigned to bacteria in the vitreous fluid of control (VC) and postfever retinitis (PFR) individuals groups.

Samples	Total/ Average	Reads in Millions (Q > 25)	Reads Assigned to Bacteria
VC	Total	79.24	858,444
	Average	4.17	45,181.3
PFR	Total	47.4	282,642
	Average	5.27	31,404.7
VC + PFR	Total	126.64	1,141,086
	Average	4.52	40,753

**Table 2:** Discriminative genera in VC and PFR groups (*p* < 0.05).

Genera	VC *	PFR *	*p* Value (Wilcoxon)
*Paenibacillus*	3.466785	2.272994	0.002
*Bacillus*	1.423273	0.941839	0.032
*Propionibacterium*	0.567522	0.116189	0.038
*Pelosinus*	0.278704	0.159072	0.048
*Shigella*	0.096847	0.042937	0.041
*Sediminibacterium*	0.094356	0.028078	0.033
*Janthinobacterium*	0.050997	0.018052	0.019
*Thermosinus*	0.040074	0.012004	0.022
*Bdellovibrio*	0.03733	0.0074	0.005
*Acetonema*	0.034731	0.010722	0.024
*Sporomusa*	0.035308	0.006305	0.028
*Mesorhizobium*	0.030874	0.004932	0.003
*Anaerotruncus*	0.02709	0.009003	0.048
*Geobacillus*	0.018645	0.004591	0.038
*Shimwellia*	0.011266	0	0.011
*Arthrobacter*	0.008512	0	0.045
*Tannerella*	0	0.00896	0.042
*Pimelobacter*	0	0.002584	0.01

* Mean abundance.

**Table 3:** Functional characteristics of the discriminative bacterial genera that were differentially abundant in the vitreous fluid of control (VC) group and postfever retinitis (PFR) group.

Genera	Function	Abundance Increased or Decreased in Vitreous of VC or PFR	References
*Acetonema*	Unknown	Decreased in PFR	-
*Anaerotruncus*	Probiotic	Decreased in PFR	[40]
*Arthrobacter*	Probiotic	Decreased in PFR	[8]
*Bacillus*	Antimicrobial peptides	Decreased in PFR	[41]
*Bdellovibrio*	Antimicrobial	Decreased in PFR	[42]
*Geobacillus*	Unknown	Decreased in PFR	-
*Janthinobacterium*	Antibacterial, fungal and viral (violacin)/Probiotic	Decreased in PFR	[43]
*Mesorhizobium*	Unknown	Decreased in PFR	-
*Paenibacillus*	Antimicrobial lipopeptides	Decreased in PFR	[44]
*Pelosinus*	Unknown	Decreased in PFR	-
*Pimelobacter*	Unknown	Increased in PFR	-
*Propionibacterium*	Proinflammatory	Decreased in PFR	[38]
*Sediminibacterium*	Unknown	Decreased in PFR	-
*Shigella*	Proinflammatory	Decreased in PFR	[45]
*Shimwellia*	Probiotic	Decreased in PFR	[46]
*Sporomusa*	Antimicrobial and anti-inflammatory	Decreased in PFR	[47]
*Tannerella*	Proinflammatory	Increased in PFR	[34]
*Thermosinus*	Unknown	Decreased in PFR	-

## Supplementary Materials

Supplementary materials can be found at https://www.mdpi.com/2076-2607/8/5/751/s1. Figure S1: Rarefaction curves of the bacterial microbiomes generated for the 28 vitreous samples collected from healthy controls (VC) and postfever retinitis (PFR) individuals. Figure S2: A. Venn diagram illustrating the shared genera in the vitreous of VC and PFR individuals. B. Correlation network of 18 discriminative genera in VC and PFR individuals. Genera having a mean abundance > 0.002% were analysed. Table S1. Details of vitreous fluid collected from control (VC, *n* = 19) and postfever retinitis individuals (PFR, *n* = 9) Table S2: Abundance of bacterial phyla in the vitreous of healthy controls (VC) and postfever retinitis (PFR) individuals. Table S3A: Bacterial biome table of the vitreous of healthy controls (VC) and postfever retinitis (PFR) individuals. Table S3B: Abundance of bacterial genera in the vitreous of healthy controls (VC) and postfever retinitis (PFR) individuals. Genera having a mean abundance of >0.002% are listed in the table. Table S4: Co-occurrence network analysis of the genera in the vitreous fluid of healthy controls (VC) (A) and postfever retinitis (PFR) individuals (B). Table S5: Discriminative KEGG pathways in VC and PFR groups (*p* < 0.05).
