# Supplementary material for: Comparison of the Vitreous Fluid Bacterial Microbiomes between Individuals with Post Fever Retinitis and Healthy Controls
Source: Microorganisms. 2020 May 17;8(5):751. doi: 10.3390/microorganisms8050751 (PMC7285296; doi:10.3390/microorganisms8050751)
Supplement: Supplementary file 1 [file microorganisms-08-00751-s001.zip › microorganisms-766717-new/Supplementary_Tables_revision1.pdf]

Table S1. Detaills of control samples (VC, n=19) and post fever retinitis samples (PFR, n=9)

Sl. No.	Sample ID	Age (years)	Gender	State	Vitreous sample collection procedure	History of Retinitis
1	VC01	61	Female	Telangana	Pars plana vitrectomy	nil
2	VC02	46	Female	Karnataka	Pars plana vitrectomy	nil
3	VC03	63	Male	Madhya Pradesh	Pars plana vitrectomy	nil
4	VC04	47	Male	West Bengal	Pars plana vitrectomy	nil
5	VC05	36	Male	Andhra Pradesh	Pars plana vitrectomy	nil
6	VC06	36	Male	Andhra Pradesh	Pars plana vitrectomy	nil
7	VC07	22	Male	West Bengal	Pars plana vitrectomy	nil
8	VC08	57	Male	Telangana	Pars plana vitrectomy	nil
9	VC09	67	Female	Andhra Pradesh	Pars plana vitrectomy	nil
10	VC10	69	Male	Maharashtra	Pars plana vitrectomy	nil
11	VC11	54	Male	Telangana	Pars plana vitrectomy	nil
12	VC12	56	Male	Telangana	Pars plana vitrectomy	nil
13	VC13	37	Male	Telangana	Pars plana vitrectomy	nil
14	VC14	33	Female	Andhra Pradesh	Pars plana vitrectomy	nil
15	VC15	68	Female	Telangana	Pars plana vitrectomy	nil
16	VC16	42	Male	Telangana	Pars plana vitrectomy	nil
17	VC17	44	Male	Andhra Pradesh	Pars plana vitrectomy	nil
18	VC18	44	Male	Andhra Pradesh	Pars plana vitrectomy	nil
19	VC19	31	Male	Andhra Pradesh	Pars plana vitrectomy	nil
20	PFR01	35	Female	Maharashtra	Pars plana vitrectomy	Post febrile illness
21	PFR02	32	Male	Telangana	Vitreous biopsy	Post Typhoid fever
22	PFR03	20	Female	Karnataka	Vitreous biopsy	Post febrile illness
23	PFR04	42	Male	Telangana	Vitreous biopsy	Post febrile illness
24	PFR05	39	Male	Telangana	Vitreous biopsy	Post Typhoid fever
25	PFR06	40	Female	Andhra Pradesh	Vitreous biopsy	Post febrile illness
26	PFR07	46	Male	Telangana	Pars plana vitrectomy	Taxoplasma retinitis
27	PFR08	26	Male	Telangana	Pars plana vitrectomy	Tuberculosis retinitis

28	PFR09	46	Male	Telangana	Vitreous biopsy	Viral retinitis

Table S2. Abundance of bacterial phyla in the vitreous of Control (VC) and post fever retinitis (PFR) samples

Sl. No	Phyla	Phyla Abundance in VC		Phyla Abundance in PFR	
		Mean	Range	Mean	Range
1	*Actinobacteria*	1.652731	0.124- 5.844	1.40761	0.092- 4.28
2	*Aquificae*	0.000128	0-0.0024	0	0
3	*Armatimonadetes*	0	0	0.000528	0- 0.0047
4	*Bacteroidetes*	5.056217	3.293-7.030	8.747335	2.536-44.575
5	*Caldiserica*	0	0	0.000528	0- 0.0047
6	*Verrucomicrobia*	0.962347	0-5.180	1.448536	0.014-5.985
7	*Deferribacteres*	0	0	0.005275	0-0.475
8	*Deinococcus-Thermus*	0.005109	0-0.0448	0.005239	0-0.0191
9	*Acidobacteria*	0.007881	0-0.0872	0.002127	0-0.0191
10	*Firmicutes*	44.51815	14.730-57.154	36.30652	8.500-63.462
11	*Fusobacteria*	0.143973	0.044-0.638	0.137362	0.038-0.122
12	*Nitrospira*	0	0	0.001055	0-0.00949
13	*Planctomycetes*	0.206759	0-2.704	0.480319	0-1.997
14	*Proteobacteria*	46.5706	36.196-78.514	50.3268	29.053-88.782
15	*Spirochaetes*	0.094064	0-0.232	0.072738	0-0.212
16	*Synergistetes*	0.002445	0-0.284	0	0
17	*Tenericutes*	0.415196	0.035-1.506	0.640988	0.0185-1.752
18	*Thermotogae*	0.00317	0-0.0024	0.002655	0-0.0026
19	unclassified	0.295438	0-0.788	0.386172	0-1.760

Table S3B. Abundance of bacterial genera in the vitreous of healthy controls (VC) and post fever retinitis (PFR) individuals. Genera having a mean abundance of >0.002% are listed in the table.

Sl. No	Genera	Mean abundance VC	Mean abundance PFR
1	*Acetivibrio*	0.027185	0.016641
2	*Acetobacter*	0.005039	0.005583
3	*Acetobacterium*	0.048813	0.031659
4	*Acetonema*	0.034864	0.010727
5	*Acholeplasma*	0.009875	0.004596
6	*Achromobacter*	0.043354	0.072312
7	*Acidaminococcus*	0.002692	0.001055
8	*Acidovorax*	0.034869	0.058179
9	*Acinetobacter*	7.869491	6.889979
10	*Actinobacillus*	0.003302	0.003165
11	*Actinomyces*	0.019501	0.000528
12	*Aeromonas*	0.017118	0.01148
13	*Afipia*	0.002295	0.004528
14	*Alcanivorax*	0.529653	0.806862
15	*Alicycliphilus*	0.003337	0.001055
16	*Alicyclobacillus*	0.001524	0.006065
17	*Alishewanella*	0.004591	0
18	*Alistipes*	0.001497	0.011605
19	*Alkaliphilus*	0.052207	0.038822
20	*Allofustis*	0.003006	0.001691
21	*Anaerococcus*	0.004619	0.004961
22	*Anaerosalibacter*	0.010027	0.004055
23	*Anaerostipes*	0.026541	0.042545
24	*Anaerotruncus*	0.027115	0.009004
25	*Aneurinibacillus*	0.010051	0.005959
26	*Anoxybacillus*	0.006951	0.002834
27	*Aquimarina*	0.005251	0.002127
28	*Arcobacter*	0.004591	0

29	*Arcticibacter*	0.010836	0.002768
30	*Arthrobacter*	0.00863	0
31	*Atopobium*	0.018787	0.005064
32	*Azospirillum*	0.003101	0.005248
33	*Bacillus*	1.430659	0.946169
34	*Bacteroides*	0.421354	2.447203
35	*Barnesiella*	0	0.006858
36	*Bdellovibrio*	0.037541	0.007405
37	*Beggiatoa*	0.323671	0.590769
38	*Bifidobacterium*	0.017643	0.080112
39	*Bilophila*	0.009181	0.020238
40	*Blautia*	0.247408	0.207441
41	*Bordetella*	0.916007	2.137875
42	*Borrelia*	0.007045	0.014295
43	*Bosea*	0.003012	0.000528
44	*Brachyspira*	0.004336	0.003595
45	*Bradyrhizobium*	0.031089	0.016484
46	*Brevibacillus*	0.052829	0.03079
47	*Brevundimonas*	0.019854	0.030011
48	*Brucella*	0.006921	0.002584
49	*Bryobacter*	0.00329	0
50	*Burkholderia*	0.138859	0.083921
51	*Buttiauxella*	0.017137	0.006895
52	*Butyricicoccus*	0.0061	0.008001
53	*Butyrivibrio*	0.19192	0.13975
54	*Caldicellulosiruptor*	0.019506	0.014411
55	*Caldicoprobacter*	0.012515	0.00877
56	*Caloramator*	0.034792	0.021824
57	*Caloranaerobacter*	0.007512	0.004977
58	*Campylobacter*	0.800037	1.381903
59	*Capnocytophaga*	0.003232	0.004021

60	*Carnobacterium*	0.058124	0.021387
61	*Catonella*	0.022015	0.022823
62	*Cedecea*	0.022626	0.020947
63	*Cellulosilyticum*	0.051843	0.041877
64	*Chitinophaga*	0.017575	0.006657
65	*Chlamydia*	0.942833	1.420587
66	*Chondromyces*	0.005357	0.002127
67	*Chryseobacterium*	0.995588	0.63926
68	*Citrobacter*	1.65672	1.351335
69	*Clostridiisalibacter*	0.001902	0.005203
70	*Clostridium*	19.47415	15.37239
71	*Cohnella*	0.034084	0.014293
72	*Collinsella*	0	0.00633
73	*Comamonas*	0.083524	0.032655
74	*Coprobacillus*	0.015625	0.016024
75	*Coprococcus*	0.061077	0.036433
76	*Corynebacterium*	0.117906	0.037441
77	*Cronobacter*	0.420222	0.203246
78	*Cupriavidus*	0.011232	0.001583
79	*Curtobacterium*	0.007652	0.012247
80	*Curvibacter*	0.0099	0.002349
81	*Cytophaga*	0.004591	0
82	*Dehalobacter*	0.020024	0.012992
83	*Dehalobacterium*	0.002499	0.008281
84	*Deinococcus*	0.004981	0.002584
85	*Delftia*	0.130036	0.167493
86	*Desulfitibacter*	0.001495	0.003665
87	*Desulfitobacterium*	0.200427	0.147917
88	*Desulfococcus*	0.002295	0.004528
89	*Desulfosporosinus*	0.166438	0.097347
90	*Desulfotomaculum*	0.05783	0.039292

91	*Desulfovibrio*	0.009485	0.040986
92	*Dialister*	0.000746	0.060137
93	*Dickeya*	0.02113	0.017435
94	*Dielma*	0.006266	0.00668
95	*Dorea*	0.066814	0.048661
96	*Dyadobacter*	0.01056	0.013423
97	*Dysgonomonas*	0.004827	0.006655
98	*Edwardsiella*	0.01664	0.017286
99	*Elizabethkingia*	0.039928	0.023689
100	*Empedobacter*	0.007353	0.005746
101	*Enhydrobacter*	0.120521	0.043626
102	*Enterobacter*	10.31277	10.39126
103	*Enterococcus*	0.166025	0.144036
104	*Epilithonimonas*	0.009301	0.001999
105	*Erwinia*	0.041441	0.02234
106	*Erysipelatoclostridium*	0.010626	0.006643
107	*Erysipelothrix*	0.001474	0.006751
108	*Erythrobacter*	0.004316	0.004528
109	*Escherichia*	1.572377	1.642283
110	*Ethanoligenens*	0.010856	0.008282
111	*Eubacterium*	0.302752	0.438006
112	*Exiguobacterium*	0.005571	0.001845
113	*Faecalibacterium*	0.01874	0.086227
114	*Fervidicella*	0.036965	0.015798
115	*Fictibacillus*	0.002907	0.001384
116	*Finegoldia*	0.010748	0.002655
117	*Flavihumibacter*	0.016994	0.004528
118	*Flavobacterium*	0.091794	0.049063
119	*Flavonifractor*	0.000904	0.100756
120	*Flexithrix*	0.003623	0
121	*Fusobacterium*	0.133512	0.130794

122	*Gardnerella*	0.007106	0.002127
123	*Gemmata*	0.204464	0.479264
124	*Geobacillus*	0.018659	0.004592
125	*Geobacter*	0.002455	0.001583
126	*Gilvimarinus*	0.002295	0.004528
127	*Gordonia*	0.004279	0.005583
128	*Gottschalkia*	0.024369	0.013551
129	*Haemophilus*	0.021359	0.003112
130	*Hafnia*	0.011029	0.008223
131	*Halomonas*	0.011904	0.009378
132	*Haloplasma*	0.004118	0.001384
133	*Helicobacter*	0.913535	1.341049
134	*Heliobacterium*	0.013475	0.007719
135	*Herbaspirillum*	0.040173	0.204362
136	*Herminiimonas*	0.00342	0.003639
137	*Histophilus*	0	0.033761
138	*Holdemania*	0.003977	0.002306
139	*Intestinibacter*	0.001932	0.002153
140	*Intestinimonas*	0.010331	0.016175
141	*Janthinobacterium*	0.051254	0.018062
142	*Jeotgalibacillus*	0.006884	0.001845
143	*Klebsiella*	5.996647	7.493745
144	*Kluyvera*	0.033795	0.020796
145	*Kosakonia*	0.059333	0.050283
146	*Lachnoanaerobaculum*	0.026541	0.018757
147	*Lachnoclostridium*	7.097401	5.047334
148	*Lachnospira*	0.001468	0.007827
149	*Lactobacillus*	0.463949	0.487873
150	*Lactococcus*	0.030436	0.026828
151	*Laribacter*	0	0.031651
152	*Leclercia*	1.17409	1.01749

153	*Legionella*	0.003294	0.005055
154	*Lelliottia*	0.026789	0.07305
155	*Leptotrichia*	0.002926	0.004568
156	*Leuconostoc*	0.297039	0.146516
157	*Listeria*	0.847872	1.277845
158	*Lysinibacillus*	1.344975	0.81703
159	*Lysobacter*	0.000904	0.004528
160	*Magnetospirillum*	0.003157	0.000528
161	*Mahella*	0.013064	0.009911
162	*Marinobacterium*	0.002295	0.002057
163	*Marinomonas*	0.005342	0.004528
164	*Marvinbryantia*	0.027236	0.016123
165	*Massilia*	0.010194	0.001583
166	*Megamonas*	0.002763	0.005695
167	*Megasphaera*	0.010457	0.116163
168	*Mesorhizobium*	0.031111	0.004934
169	*Methylobacterium*	0.006248	0.003112
170	*Methylosinus*	0.00235	0.002057
171	*Microbacterium*	0.252861	0.34545
172	*Microbulbifer*	0	0.007913
173	*Moraxella*	0.009421	0.008387
174	*Morganella*	0.00668	0.005168
175	*Moritella*	0.00561	0
176	*Mucilaginibacter*	0.013842	0.005098
177	*Muricauda*	0	0.027958
178	*Mycobacterium*	0.27267	0.249663
179	*Mycoplasma*	0.007607	0.006078
180	*Myroides*	0.012356	0.005884
181	*Neisseria*	0.771574	1.5235
182	*Niabella*	0.016347	0.007077
183	*Niastella*	0.037129	0.001999

184	*Nitrosospira*	0.00392	0
185	*Nocardia*	0.011697	0
186	*Novosphingobium*	0.003238	0.05001
187	*Oceanobacillus*	0.007713	0.004362
188	*Ochrobactrum*	0.118098	0.086106
189	*Olivibacter*	0.015636	0.015256
190	*Oribacterium*	0.024825	0.021085
191	*Ornithinibacillus*	0.004574	0.015391
192	*Ornithobacterium*	0.002992	0.0084
193	*Oscillibacter*	0.075247	0.045256
194	*Paenibacillus*	3.493438	2.295071
195	*Paenisporosarcina*	0.006922	0.001691
196	*Pantoea*	0.101955	0.074905
197	*Parabacteroides*	0.01181	0.23683
198	*Paracoccus*	0.005571	0.002584
199	*Paucisalibacillus*	0.003738	0.002127
200	*Pectobacterium*	0.391466	0.545898
201	*Pediococcus*	0.150993	0.433683
202	*Pedobacter*	0.196315	0.085537
203	*Pelosinus*	0.279775	0.159687
204	*Peptoclostridium*	0.118144	0.074182
205	*Peptoniphilus*	0.029837	0.003229
206	*Phascolarctobacterium*	0.003824	0
207	*Photobacterium*	0.009479	0.004774
208	*Photorhabdus*	0.03613	0.029234
209	*Phyllobacterium*	0.028973	0.013599
210	*Pimelobacter*	0	0.007112
211	*Piscirickettsia*	0.285943	0.480366
212	*Planococcus*	0.000897	0.006224
213	*Pluralibacter*	0.004194	0.005749
214	*Porphyrobacter*	0.011184	0

215	*Porphyromonas*	0.059433	0.044388
216	*Prevotella*	0.143286	2.703511
217	*Propionibacterium*	0.574039	0.116949
218	*Proteiniclasticum*	0.006708	0.012072
219	*Proteiniphilum*	0	0.006858
220	*Proteocatella*	0.010599	0.002306
221	*Providencia*	0.004382	0.00344
222	*Pseudoalteromonas*	0.698673	0.996129
223	*Pseudobacteroides*	0.020555	0.014014
224	*Pseudobutyrivibrio*	0.011034	0.002614
225	*Pseudoflavonifractor*	0.019382	0.009077
226	*Pseudomonas*	6.37975	5.984171
227	*Psychrobacter*	0.004484	0.002439
228	*Rahnella*	0.052727	0.027546
229	*Ralstonia*	0.014897	0.011973
230	*Raoultella*	0.050642	0.06015
231	*Rheinheimera*	0.020416	0
232	*Rhodococcus*	0.029627	0.009239
233	*Rhodospirillum*	0.003313	0.004528
234	*Rickettsieae*	0.008603	0
235	*Robinsoniella*	0.146613	0.090229
236	*Roseburia*	0.206988	0.158405
237	*Rothia*	0.003962	0
238	*Rufibacter*	0.000192	0.008968
239	*Ruminiclostridium*	0.600838	0.396195
240	*Ruminococcus*	0.19929	0.180849
241	*Runella*	0.006823	0.000528
242	*Saccharibacillus*	0.012364	0.009681
243	*Saccharibacter*	0.004488	0
244	*Salinibacillus*	0.008944	0.008321
245	*Salinispora*	0.001496	0.004528

246	*Salmonella*	0.612725	0.562065
247	*Sebaldella*	0.007407	0.001999
248	*Sediminibacterium*	0.095013	0.028232
249	*Segetibacter*	0.008459	0
250	*Selenomonas*	0.019511	0.036788
251	*Serratia*	0.090354	0.086191
252	*Shewanella*	0.107185	0.642926
253	*Shigella*	0.097444	0.043049
254	*Shimwellia*	0.011276	0
255	*Solibacillus*	0.000833	0.004641
256	*Sphaerochaeta*	0.018799	0.015029
257	*Sphingobacterium*	2.501145	1.91209
258	*Sphingomonas*	0.011691	0.001055
259	*Sphingopyxis*	0.003952	0
260	*Spirochaeta*	0.018188	0.008711
261	*Spirosoma*	0.007841	0.004582
262	*Sporolactobacillus*	0.010791	0.006266
263	*Sporomusa*	0.035345	0.006306
264	*Sporosarcina*	0.006928	0.000528
265	*Staphylococcus*	2.732013	3.190444
266	*Stenotrophomonas*	0.662983	0.785941
267	*Streptococcus*	1.070676	1.382554
268	*Streptomyces*	0.287011	0.472153
269	*Subdoligranulum*	0.01834	0.009804
270	*Syntrophobotulus*	0.022386	0.028556
271	*Tannerella*	0	0.013496
272	*Tatumella*	0.008244	0.005064
273	*Taylorella*	0	0.007913
274	*Tepidanaerobacter*	0.002458	0.004193
275	*Terrimonas*	0.015864	0
276	*Terrisporobacter*	0.018251	0.021298

277	*Tetrasphaera*	0.002295	0.009056
278	*Thermoanaerobacter*	0.014746	0.015884
279	*Thermoanaerobacterium*	0.012357	0.016296
280	*Thermobrachium*	0.018398	0.022245
281	*Thermosinus*	0.040263	0.012052
282	*Thermotoga*	0.003042	0
283	*Trabulsiella*	0.020225	0.009932
284	*Treponema*	0.0442	0.030054
285	*Turicibacter*	0.001935	0.002746
286	*Tyzzerella*	0.015662	0.009203
287	*Variovorax*	0.012481	0.012394
288	*Veillonella*	0.009833	0.011147
289	*Verminephrobacter*	0.002295	0.002057
290	*Vibrio*	0.531316	0.720093
291	*Virgibacillus*	0.004115	0.009169
292	*Viridibacillus*	0.004666	0.001384
293	*Vitreoscilla*	0.002295	0.002057
294	*Waddlia*	0.014923	0.026893
295	*Xanthomonas*	0.155598	0.243403
296	*Xenophilus*	0.006886	0.004528
297	*Xenorhabdus*	0.008243	0.002746
298	*Yersinia*	0.078359	0.047099
299	*Yokenella*	0.052226	0.027981
300	*Youngiibacter*	0.015709	0.009496
301	*Zymophilus*	0.01117	0.008015
	Total Number	291	281

Table S4A: Co-occurrence network analysis of control (VC) group determining the number of positive and negative interactions among the genera

**Genera**	**Positive interaction**	**Negative interaction**	**Sample count (%)**
Acetonema	14	1	73.7
Anaerotruncus	14	1	63.1
Arthrobacter	14	1	42.1
Bacillus	14	1	100
Bdellovibrio	14	1	84.2
Geobacillus	14	1	68.4
Janthinobacterium	13	2	84.2
Mesorhizobium	14	1	84.2
Paenibacillus	13	2	100
Pelosinus	14	1	84.2
Propionibacterium	2	13	73.7
Sediminibacterium	15	0	78.9
Shigella	14	1	84.2
Shimwellia	13	2	57.9
Sporomusa	14	1	63.1
Thermosinus	14	1	73.7

Table S4B: Co-occurrence network analysis of PFR group determining the number of positive and negative interactions among the genera

**Genera**	**Positive interaction**	**Negative interaction**	**Sample count (%)**
Acetonema	15	0	33.3
Anaerotruncus	15	0	22.2
Bacillus	13	2	88.9
Bdellovibrio	14	1	44.4
Geobacillus	13	2	22.2
Janthinobacterium	13	2	55.6
Mesorhizobium	11	4	33.3
Paenibacillus	9	6	100
Pelosinus	12	3	77.8
Pimelobacter	12	3	22.2
Propionibacterium	11	4	22.2
Sediminibacterium	15	0	33.3
Shigella	13	2	55.6
Sporomusa	15	0	22.2

Tannerella	13	2	11.1
Thermosinus	15	0	44.4

Table S5: Discriminative KEGG pathways in VC and PFR groups (P<0.05).

KEGG_Pathways	PFR*	VC*	P value (Wilcoxon)
ErbB signaling pathway	0.310609	0.136254	0.009
MAPK signaling pathway - fly	0.063988	0.039978	0.023
VEGF signaling pathway	0.122926	0.081402	0.024
PPAR signaling pathway	0.138487	0.119535	0.046
Lysine degradation	0.246942	0.393139	0.008
Tryptophan metabolism	0.188437	0.281077	0.027
Glutathione metabolism	0.206099	0.429214	0.014
Taurine and hypotaurine metabolism	0.029744	0.072595	0.003
beta-Alanine metabolism	0.121669	0.214489	0.014
C5-Branched dibasic acid metabolism	0.084834	0.17598	0.014
Pentose phosphate pathway	0.338875	0.578695	0.034
Steroid degradation	0.00425	0.000431	0.002
Flavone and flavonol biosynthesis	0.006449	0.001139	0.046
Intestinal immune network for IgA production	0.055213	0.009871	0.009

* Mean abundance.
