# Supplementary figures and images for: Comparison of the Vitreous Fluid Bacterial Microbiomes between Individuals with Post Fever Retinitis and Healthy Controls
Source: Microorganisms. 2020 May 17;8(5):751. doi: 10.3390/microorganisms8050751 (PMC7285296; doi:10.3390/microorganisms8050751)

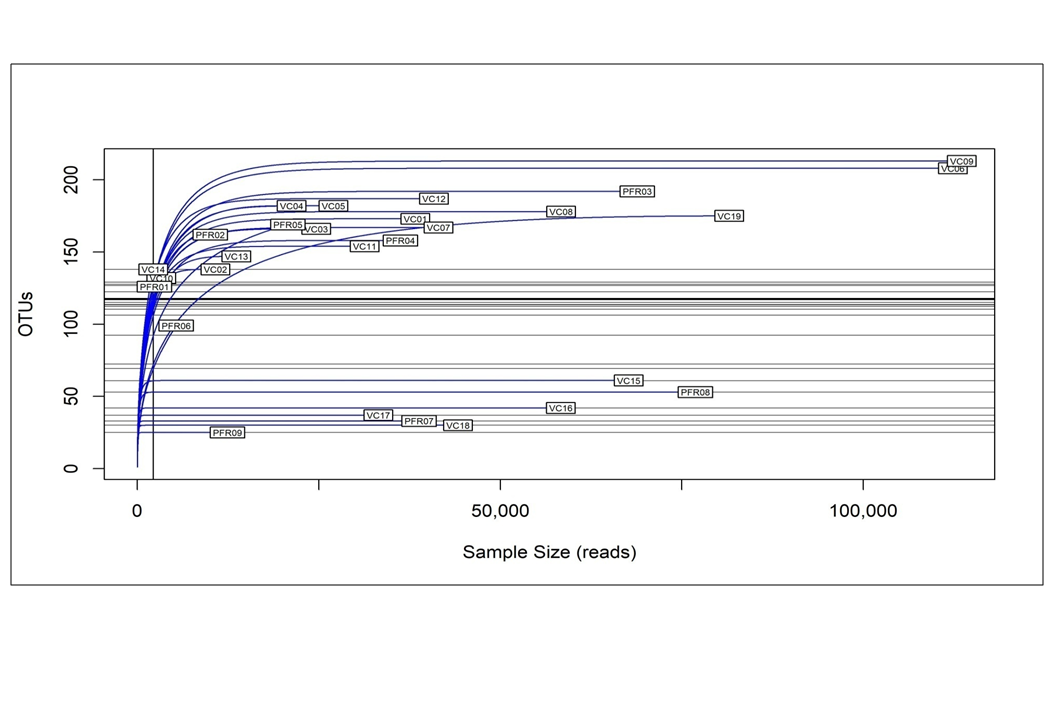

Supplement: Supplementary file 1 [file microorganisms-08-00751-s001.zip › microorganisms-766717-new/Figure S1.tif]

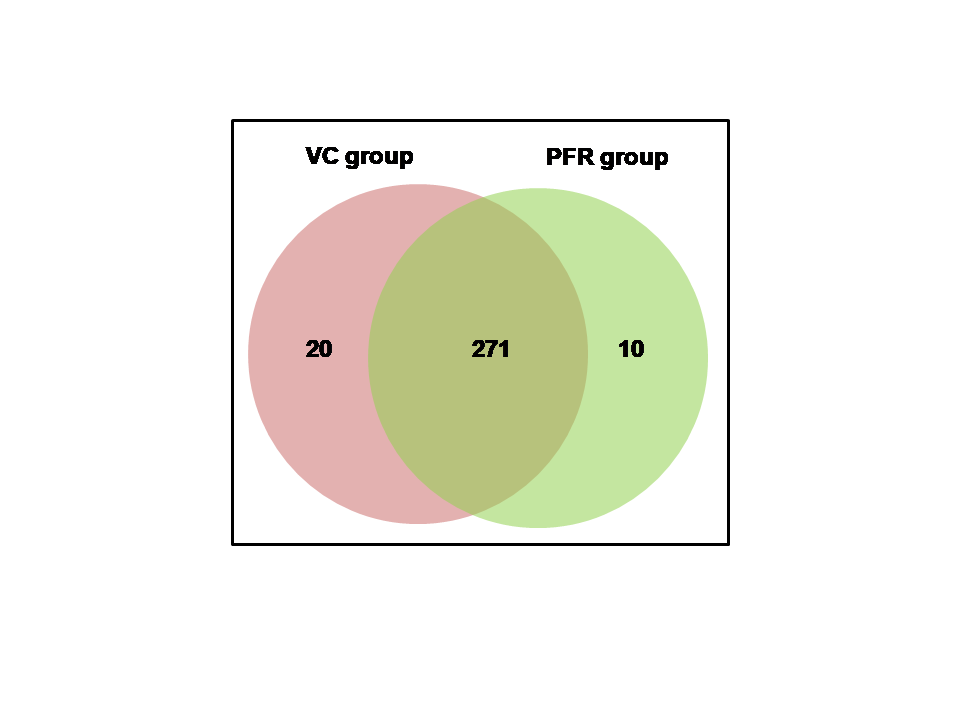

Supplement: Supplementary file 1 [file microorganisms-08-00751-s001.zip › microorganisms-766717-new/Figure S2.tif]
